# Bacterial Spore mRNA – What’s Up With That?

**DOI:** 10.3389/fmicb.2020.596092

**Published:** 2020-10-26

**Authors:** Peter Setlow, Graham Christie

**Affiliations:** ^1^Department of Molecular Biology and Biophysics, UConn Health, Farmington, CT, United States; ^2^Department of Chemical Engineering and Biotechnology, University of Cambridge, Cambridge, United Kingdom

**Keywords:** *Bacillus*, spores, mRNA, germination, sporulation

## Abstract

Bacteria belonging to the orders Bacillales and Clostridiales form spores in response to nutrient starvation. From a simplified morphological perspective, the spore can be considered as comprising a central protoplast or core, that is, enveloped sequentially by an inner membrane (IM), a peptidoglycan cortex, an outer membrane, and a proteinaceous coat. All of these structures are characterized by unique morphological and/or structural features, which collectively confer metabolic dormancy and properties of environmental resistance to the quiescent spore. These properties are maintained until the spore is stimulated to germinate, outgrow and form a new vegetative cell. Spore germination comprises a series of partially overlapping biochemical and biophysical events – efflux of ions from the core, rehydration and IM reorganization, disassembly of cortex and coat – all of which appear to take place in the absence of *de novo* ATP and protein synthesis. If the latter points are correct, why then do spores of all species examined to date contain a diverse range of mRNA molecules deposited within the spore core? Are some of these molecules “functional,” serving as translationally active units that are required for efficient spore germination and outgrowth, or are they just remnants from sporulation whose sole purpose is to provide a reservoir of ribonucleotides for the newly outgrowing cell? What is the fate of these molecules during spore senescence, and indeed, are conditions within the spore core likely to provide any opportunity for changes in the transcriptional profile of the spore during dormancy? This review encompasses a historical perspective of spore ribonucleotide biology, from the earliest biochemical led analyses – some of which in hindsight have proved to be remarkably prescient – through the transcriptomic era at the turn of this century, to the latest next generation sequencing derived insights. We provide an overview of the key literature to facilitate reasoned responses to the aforementioned questions, and many others, prior to concluding by identifying the major outstanding issues in this crucial area of spore biology.

## Prelude

When RNA species in spores of Bacillales species were first characterized more than 50 years ago, spores were found to contain both rRNAs and tRNAs, although some of these nucleic acids exhibited a few apparent differences from their growing cell counterparts (Chambon et al., 1968; Deutscher et al., 1968; Setlow et al., 1974). The existence of functional spore mRNA, however, seemed less likely, since several early studies showed that spores germinating in the presence of inhibitors of RNA polymerase made little if any detectable protein (Steinberg et al., 1965; Torriani and Levinthal, 1967; Setlow and Primus, 1975), although it is possible that RNA synthesis inhibitors may not enter the spore core until germination is complete. However, even ~50 years ago there was evidence that spores did contain some mRNA, and that some of this mRNA was translationally active, at least *in vitro* (Chambon et al., 1968; Deutscher et al., 1968; Jeng and Doi, 1974), even if this spore mRNA appeared to be non-functional during spore germination and subsequent outgrowth. However, the thinking about spore mRNA was changed dramatically beginning ~14 years ago in the transcriptomics revolution, as over the following years a number of laboratories found 100s to 1,000s of specific mRNAs in spores of a large number of Bacillales as well as Clostridiales species (Bergman et al., 2006; Bettegowda et al., 2006; Keijser et al., 2007; Jones et al., 2008; van Melis et al., 2011; Nicholson et al., 2012; Segev et al., 2012; Bassi et al., 2013; Dembek et al., 2013; Bate et al., 2014; Swarge et al., 2018). This review will trace the history of spore mRNA biology and summarize the more recent work, some of which is ongoing. The focus will be on what we do know, and more importantly what we do not know about spore mRNA, including how these mRNAs come to be present in dormant spores. Perhaps most significantly, we will also consider the function of the plethora of mRNAs in dormant bacterial spores, and, just as importantly, what this function is not.

## Introduction

**Figure 1:**
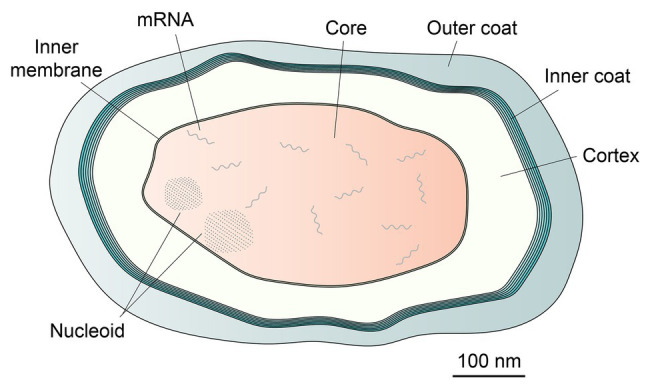
Schematic of a dormant *Bacillus subtilis* spore. Spores of all species share similar morphological features, namely a coat, which in this species can be sub-divided into distinct inner and outer layers, a peptidoglycan cortex, and a membrane bound spore core. The nucleoid comprises DNA encrusted by protective SASP proteins, visible as clusters of ordered dots. mRNA content is present only within the spore core.

Spores are formed by some Firmicutes species, the most well-studied being those of the Bacillales, although studies on spores of Clostridiales species are increasing (Tan and Ramamurthi, 2014; Setlow and Johnson, 2019; Shen et al., 2019). Such spores are considered metabolically dormant and extremely resistant to all manner of potentially harmful treatments, including wet and dry heat, desiccation, high radiation levels, and a host of toxic chemicals, including antibiotics, and can survive for many years (Setlow, 2006; Ulrich et al., 2018). Notably, dormant spores’ central core (Figure 1), comparable to the protoplast of a growing cell, has an extremely low water content, as low as 25% of wet wt in spores of some thermophiles and 35% of wet wt in the well-studied *Bacillus subtilis* spores; this is in contrast to the value of 80% of wet wt as water in growing cells or fully germinated spores (Gerhardt and Marquis, 1989). Spores have been studied in part because of their fascinating life cycle of growth, sporulation, and spore germination (Tan and Ramamurthi, 2014; Setlow et al., 2017; Shen et al., 2019). In addition, spores of a number of species are vectors for human diseases or intoxications, as well as food spoilage and food-borne disease. Consequently, there is also much applied interest in spore resistance and germination, the latter largely because a germinated spore has lost the high resistance of the dormant spore, and is easy to kill (Setlow and Johnson, 2019; Buhr et al., 2020). Together, both the basic science and applied interests in spores’ formation, properties, and germination has made this one of the best studied developmental system in biology, with definitive work going back many years.

### The Early Years

Given the technology available for use 50-plus years ago, much of early work on spores was descriptive (Setlow et al., 2017; Setlow and Johnson, 2019). This work involved: (i) isolation and mapping of sporulation mutants in *B. subtilis* because some strains of this species are naturally transformable (this is also the major reason that *B. subtilis* became the “model” spore former), (ii) characterizing biochemical and morphological events in sporulation and spore germination and their timing, including changes in metabolism, (iii) characterization and quantitation of the components in the dormant spore, including nucleic acids and small molecules, and using spores of multiple species, and (iv) examining early events in spore germination, including the release of small molecules, degradation of a specific peptidoglycan layer unique to spores, termed the cortex (Figure 1), followed by a return to metabolism and macromolecular synthesis in outgrowth, which converts a germinated spore into a growing cell (although enzyme activity in the spore core could well resume before completion of spore germination – see section Issues for the Future). Notably, one of the unique features of spores of all Bacillales and Clostridiales is the presence in the spore core of ~25% of core dry wt as CaDPA, a 1:1 chelate of Ca^2+^ and pyridine-2,6-dicarboxylic acid (dipicolinic acid, DPA; Gerhardt and Marquis, 1989). DPA is made in the mother cell compartment of the sporulating cell, and imported into developing spores probably as CaDPA *via* several spore-specific channels (Ramírez-Guadiana et al., 2017; Setlow and Johnson, 2019); this incorporation almost certainly requires energy and lowers the spore core water content appreciably. CaDPA also has roles in spore resistance, is rapidly released during spore germination, and has a signaling role in germination of spores of most, but probably not all Firmicutes (Setlow et al., 2017; Shen et al., 2019).

Among the important observations made in this early period of spore research was the characterization of spore nucleic acids, most thoroughly in *Bacillus megaterium*, an attractive subject for biochemical work because it sporulates profusely, its spores are easily purified and these spores germinate very rapidly (Levinson and Hyatt, 1966). Early analyses of nucleic acids found that spores contained the expected rRNAs, in some cases with minor modifications, and also tRNAs (Chambon et al., 1968; Deutscher et al., 1968). However, the 3' terminal A residue almost always present in the tRNA in vegetative cells is absent from approximately one third of total spore tRNAs and some adjacent C residues are also absent (Setlow, 1974). Several researchers also found a small amount of RNA in spores (2–4% of the total), which was larger than tRNA but was not small rRNA (Chambon et al., 1968; Deutscher et al., 1968; Jeng and Doi, 1974). One of the latter reports showed that this RNA hybridized throughout the spore genome, and another reported that this RNA directed a small amount of protein synthesis *in vitro*. This latter RNA was thus suggested to be mRNA or “mRNA-like.” It was also clear from the early work that tRNA in spores is minimally aminoacylated, if at all, although aminoacyl-tRNA synthetases are present (Deutscher et al., 1968; Setlow, 1974).

In addition, studies of small molecules in spores ~50 years ago found that while spores do have the coenzymes generally found in all growing cells such as FAD, NAD, NADP, and the four common ribo and deoxyribonucleotides, these are all in what might be termed a “low energy state,” with minimal if any NADH, NADPH, or ATP (Setlow and Kornberg, 1970a,b; Setlow and Setlow, 1977). Indeed, the absence of ATP (≤1% of the total adenine nucleotide pool) from spores shown ~50 years ago by chromatography of extracts from ^32^P-labeled spores and enzymatic assays using firefly luciferase, was confirmed more recently using ^31^P-NMR analysis of spore extracts (Ghosh et al., 2015). The latter assays were also carried out on spores incubated at physiological temperatures with care taken to prevent spore germination using appropriate mutant strains, and again with no ATP detected. A major conclusion from this older and newer work is that dormant spores in water have an energy charge [(ATP) + 0.5(ADP)/(ATP) + (ADP) + (AMP)] of ≤0.1, while in growing cells of both prokaryotes and eukaryotes’ energy charge is maintained at ~0.9 (Chapman et al., 1971). The ratio of ATP relative to the total adenine nucleotide pool in the dormant spore is thus ≤0.01, while this is normally at 0.8 in growing cells. This low ratio would in all likelihood make normal biosynthetic reactions using ATP thermodynamically unfavorable in the dormant spore core, leaving aside the role of the low core water content that most likely greatly restrains protein movement and enzymatic activity there also (Gerhardt and Marquis, 1989; Cowan et al., 2004).

Another set of important observations made >40 years ago was that dormant spores of *B. megaterium* did not contain one or more key enzymes for the synthesis of ribonucleotides or amino acids (Setlow and Kornberg, 1970b; Setlow and Primus, 1975). As a consequence, despite synthesis of RNA and proteins beginning relatively soon after spores were triggered to germinate, there was no detectable *de novo* synthesis of ribonucleotides or amino acids at this time, and biosynthesis of these small molecules only began long after germination was complete and then only after the required biosynthetic enzymes were synthesized during spore outgrowth. Thus, any synthesis of RNA or proteins occurring soon after the triggering of spore germination must come from spores’ reserves of ribonucleotides or amino acids, respectively. Indeed, spores contain a large amount of protein, classified as small, acid-soluble spore proteins (SASP), that are degraded soon after germination is initiated, and this provides amino acids for new protein synthesis at this time (Setlow, 2007). The energy for the earliest RNA and protein synthesis after spore germination is initiated, as well as tRNA repair, can come from the environment, if available, and also by utilizing spores’ endogenous energy reserves, including 3-phosphoglyceric acid (3PGA) as well as degradation of some amino acids generated by proteolysis (Nelson and Kornberg, 1970b; Setlow and Kornberg, 1970a; Setlow and Primus, 1975). There were also suggestions in this early period of spore research that ribonucleotides needed for RNA synthesis soon after triggering spore germination would also come from breakdown of spore RNAs (Setlow and Kornberg, 1970b), but the specific RNA degraded was not definitively identified.

Notably, the absence of nucleotide biosynthetic enzymes from spores and that these proteins are synthesized at various times in spore outgrowth have also recently been shown for *B. subtilis* spores in several proteomic studies (Swarge et al., 2018, 2020b). The latter findings were also made for amino acid biosynthetic enzymes as well. Other important observations (Nelson and Kornberg, 1970b; Setlow and Kornberg, 1970a,b; Scott and Ellar, 1978) made early in biochemical analyses of spore germination were that: (i) ATP accumulation began only after germination was completed or very near completion, (ii) catabolism of dormant spores’ endogenous energy reserves, most notably 3PGA, could support almost all ATP needed for at least the first few minutes after germination was triggered, and (iii) even if ATP accumulation was blocked, germination still took place. This latter observation was also confirmed more recently (Cowan et al., 2004). The thinking about mRNA in spores was also greatly influenced by experiments showing that spores of several species incubated with germinants in the presence of inhibitors of RNA synthesis still germinated, but protein synthesis was blocked >99% (Steinberg et al., 1965; Torriani and Levinthal, 1967; Setlow and Kornberg, 1970b). While this certainly does not prove that spores have no mRNA, it does suggest that the amount of functional mRNA in spores was either minimal, or this mRNA was very rapidly degraded early in spore germination.

Overall, based on the evidence available in the early years of biochemical studies on dormant spores and spore germination, the general thinking was that: (i) there was minimal if any ATP in spores and therefore minimal if any metabolic activity, (ii) this latter statement included even metabolism of endogenous energy reserves such as 3PGA – note that enzymes for converting 3PGA to acetate, NADH, and ATP are present in spores and generate ATP early after spore germination is initiated, but 3PGA is stable in dormant spores (Nelson and Kornberg, 1970a), and this latter point was revisited recently and confirmed by ^31^P-NMR of extracts from spores stored at physiological temperatures for weeks (Ghosh et al., 2015), (iii) there is neither functional mRNA nor ribonucleotide biosynthetic enzymes in dormant spores, and therefore synthesis of protein when spores germinate will require synthesis of new mRNA using ribonucleotides generated by degradation of some dormant spore RNA. Indeed, there was indirect evidence for this RNA degradation even 50 years ago (Setlow and Kornberg, 1970a,b), as *B. megaterium* spores germinating with glucose plus an inhibitor of RNA synthesis accumulated ATP levels >5-fold higher than did spores germinating with glucose alone, and (iv) spores contained ribosomes, the components of which appeared to be indistinguishable from their vegetative cell counterparts (Chambon et al., 1968; Deutscher et al., 1968). Moreover, spore ribosomes appeared to be translationally active, in the sense that when extracted, they were capable of synthesizing polyphenylalanine from a polyuracil template in cell-free reaction mixtures at comparable rates to ribosomes purified from log-phase vegetative cells (Chambon et al., 1968; Deutscher et al., 1968). Whether ribosome hibernation factors, such as the Hpf protein, have a role in spores in preserving the structural integrity of ribosomes during dormancy, analogous to that observed in stationary phase vegetative cells (Feaga et al., 2020), perhaps facilitating rapid resumption of activity upon germination, has not been established. Regardless, and most importantly, the thinking about spores until ~2006 was that they did not have “functional” mRNA.

### The Omics Era Arrives

**Figure 2:**
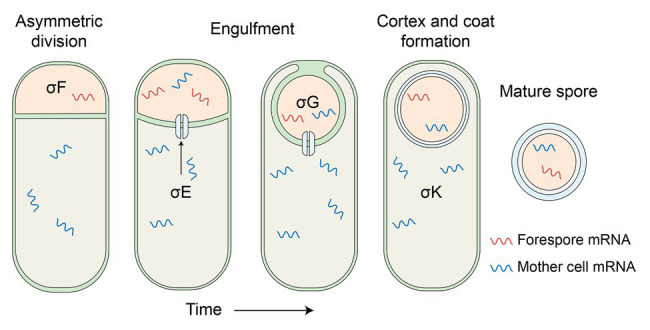
Schematic of *B. subtilis* sporulation. The process starts with an asymmetric cell division, which results ultimately in the larger mother cell compartment engulfing the nascent forespore. Both compartments are characterized by the activity of RNA polymerase containing sequentially different sporulation-specific sigma factors, the products of which progressively assemble the spore prior to its release to the environment. Despite being metabolically dormant, spores contain significant amounts of mRNA, including a minority of σE and σK associated transcripts. Neither the source nor purpose, if any, of these mother-cell associated mRNA molecules has been established, although the SpoIIIA-IIQ channel that connects both cellular compartments at or around the engulfment stage of the process may have a contributing role in the former.

The idea that spores do not have mRNA, even if this was not quite what the data from the 1960s and 1970s showed, took a real body blow in 2006 with the publication of two reports of analyses of mRNAs in bacteria of two spore formers, *B. subtilis* and *Clostridium novyi*, using microarray hybridization technology (Bettegowda et al., 2006; Keijser et al., 2007). These reports examined not only cells of these two organisms, but also dormant spores, and found readily detectable levels of mRNAs in spores from a number of genes coding for proteins ~23 in *B. subtilis* spores and 960 in *C. novyi* spores. The difference in the numbers in these two species was likely simply because of what was deemed significant in the different studies. Notably, a large number of the genes encoding mRNAs in *B. subtilis* were ones that: (i) encoded proteins known to be present in dormant spores and (ii) were expressed only in the developing forespore late in sporulation, and using RNA polymerase with the forespore-specific σ (specificity) factors for RNA polymerase, σ^F^ and/or σ^G^ (Figure 2; Arrieta-Ortiz et al., 2015; Swarge et al., 2018). The *C. novyi* spore data were more difficult to analyze fully because of the lack of proteomics data for spores of this organism. However, at least three of the most abundant five mRNAs in these spores encoded small, SASP, which in other spore formers are known to be: (i) synthesized only in the developing spore under σ^G^ and/or σ^F^ control and (ii) abundant proteins in spores (Setlow, 2007). However, note that none of these spore mRNAs have been shown to direct the synthesis of a protein after spores germinate.

The publication of the two papers cited above applying the new transcriptomics technology to spores unleashed a flood of publications reporting the mRNAs in spores of multiple species. The list of these species includes a veritable who’s who of spore formers and includes the spores of the two species noted above as well as spores of *Bacillus cereus*, *Bacillus anthracis*, *Bacillus thuringiensis*, *Clostridium acetobutylicum*, *Clostridium sporogenes*, and *Clostridioides difficile*, and several additional publications examining *B. subtilis* spores (Bergman et al., 2006; Bettegowda et al., 2006; Keijser et al., 2007; Jones et al., 2008; van Melis et al., 2011; Nicholson et al., 2012; Segev et al., 2012; Bassi et al., 2013; Dembek et al., 2013; Bate et al., 2014; Bassi et al., 2016; Nagler et al., 2016; Swarge et al., 2020a). All these studies reported generally similar findings as follows: (i) there was indeed hybridization of spore RNA to protein coding regions from genomes of these species, with these spore RNAs being almost certainly mRNAs, (ii) the variability in the numbers of these mRNAs was quite high, ranging from the low of 23 noted above to highs of >1,000, with most studies finding many 100s of mRNAs, (iii) as noted above, most of the very abundant *B. subtilis* spore mRNAs encoded proteins found in spores and their coding genes were expressed in the developing spore late in sporulation, but (iv) some mRNAs present at high levels in *B. subtilis* spores were not known to be expressed in the developing spore, and surprisingly, some were thought to be expressed only in the mother cell compartment of the sporulating cell (Figure 2). Notably, these putative mother cell mRNAs were not contaminants in outer layers of at least *B. subtilis* spores, since spores incubated in alkaline conditions which would hydrolyze RNA in spores’ outer layers still had the mother cell-expressed mRNAs present in the spore core (Korza et al., 2019).

In addition to reports of the presence of multiple mRNAs in spores, there was also extensive analysis of when the various genes encoding these spore mRNAs were actually transcribed, and whether in growing cells or during sporulation. However, these analyses were hampered by the lack of specific information on where genes were transcribed in sporulation of almost all species, in particular whether in the mother cell or forespore compartment of the sporulating cell. *B. subtilis* and *B. anthracis* were the notable exceptions to the relative paucity of definitive information on the “where” question posed above, in large part because of the classification of what σ (specificity) factors for RNA polymerase directed the transcription of various genes in developing spores (Bergman et al., 2006; Arrieta-Ortiz et al., 2015). Indeed, as noted above, of the 23 mRNAs identified in the initial report on *B. subtilis* spore mRNAs, the transcription of almost all was dependent on σ^F^ and σ^G^ known to be responsible for specific transcription late in forespore development (Keijser et al., 2007). However, for studies in which many 100s of *B. subtilis* spore mRNAs were identified, this was not the case. The second major type of analysis of the many 100s of mRNAs found in spores of different species was using one of several programs to analyze the role of the proteins encoded by the spore mRNAs in metabolism, macromolecular synthesis, and stress responses etc. (Hosack et al., 2003; Kanehisa et al., 2017). This generated a huge amount of speculation on the precise functions for specific mRNAs in spores.

A second application of the new transcriptomics technology was looking at the fate of these mRNAs in spores, and one study with *B. anthracis* spores found that they almost completely disappeared when spores were stored for ~30 days at 37°C (Bergman et al., 2006). This was largely true for *B. subtilis* spore mRNAs as well, and in even less time (Segev et al., 2012). The latter report also found that even spore rRNA was fragmented when spores were stored for various lengths of time at 37–50°C; this fragmentation also appeared to be endonucleolytic, and it was suggested to be due to RNase Y. This finding of rRNA degradation in spores incubated at elevated temperatures was confirmed more recently, and in both *B. megaterium* and *B. subtilis* spores, although not the involvement of RNase Y, and this rRNA fragmentation did not decrease spore viability or germination rates (Korza et al., 2016). However, since the rRNA fragmentation was not associated with generation of mononucleotides, it certainly appeared to be endonucleolytic. The fact that the rate of this rRNA fragmentation increased drastically as spore incubation temperature increased up to ~80°C, even though spores remained viable, suggested that the rRNA fragmentation was not enzyme catalyzed. Notably, the mRNAs in *C. novyi* spores appeared to be more stable than in spores of *Bacillus* species (Bettegowda et al., 2006), but this finding has not been studied further.

Another extremely surprising observation made based on the new technology was a report that not only was rRNA fragmented in spores incubated at 37–50°C, but that there was actual synthesis of mRNAs in spores incubated at 4°C, as shown primarily by transcriptomics and qRT-PCR (Segev et al., 2012). This report was particularly surprising in that as noted in section The Early Years above, spores have very low, if any, levels of ATP, and generate no detectable steady state level of ATP on incubation at 37°C. In addition, it seems very likely that: (i) proteins needed for RNA synthesis would not work in the low water content in the spore core and (ii) spores’ extremely low ratio of ATP/AMP would thermodynamically preclude phosphodiester bond formation, and if pyrophosphatase also does not work in the dormant spore core, this would make phosphodiester bond formation even more unfavorable. Thus, it is difficult to imagine how RNA synthesis would go on in all spores in the spore population at the same time, although it is of course impossible to prove that no spores in populations do this intermittently.

The overall picture that emerged from the work noted above in 2006–2016 was that: (i) spores appeared to have many 100s of different mRNA species, (ii) some of these spore mRNAs are synthesized specifically late in spore formation, and thus might be “left over” when the water content in the developing spore drops from 80% of wet wt to <35%, (iii) but many spore mRNAs are not known to be synthesized specifically in developing spores, and (iv) and surprisingly, some of these spore mRNAs are thought to be expressed in the mother cell compartment of the sporulating cell. So, the key questions raised but not answered by the transcriptomics work between 2006 and 2016 were: (i) where did all these various mRNAs come from and (ii) what is their function?

Some new insight into the answers to these questions posed above came from application of even newer transcriptomics technology, RNA-Seq, to analysis of *B. subtilis* spore mRNA (Nagler et al., 2016). This work found ~1,800 individual mRNAs in dormant *B. subtilis* spores, and also reported the relative abundance of these mRNAs, based on values for reads per kb of transcript per million mapped reads (RPKM). Notably, there was a huge variation in these RPKM values, ranging from >10^6^ for the most abundant mRNA down to <10 for many others, with all else in between. Analysis of these many mRNAs found that while the ~50 most abundant ones were almost all expressed in the developing spore under σ^G^ control (and see below), many spore mRNAs were known to be expressed in the mother cell compartment of the sporulating cell (Korza et al., 2019), and how such mRNAs ended up in the spore was not clear. Equally, only two of the mother-cell associated mRNA molecules were present in the top 50 or so most abundant transcripts, averaging roughly one molecule per spore (in contrast, the most abundant mRNA molecules are present at ~100 copies per spore; Korza et al., 2019). It is possible also that mother-cell associated mRNA is fragmented and may not serve as a template for translation, as discussed further below. Regardless, the results from the use of this new technology, in some ways, raised more questions than it answered. However, the improved quantitation that RNA-Seq provided over microarray hybridization, especially in terms of the whole dynamic range, certainly made the questions about these mRNAs more specific, particularly in terms of the quantitation of spore mRNAs.

### The Smoke Begins to Clear

While the reports of transcriptomics work that appeared starting in 2006 and up to 2016 provided much new information on spore mRNAs, the role of all these mRNAs was not clear. It was also not clear how all these mRNAs got into the spore core, since many were known to be synthesized in the mother cell compartment of the sporulating cell. However, the shift to analyses by RNA-Seq in the 2016 report (Nagler et al., 2016) provided important quantitative information, since the RPKM values for all ~1,800 dormant spore mRNAs identified varied over >10^5^-fold, a dynamic range that could not easily be achieved with microarray technology. Notably, of the most abundant ~50 mRNAs detected in this RNA-Seq study almost all: (i) were expressed in the developing spore late in sporulation, and under the control of σ^F^ and/or σ^G^ and (ii) encoded proteins present in the dormant spore, with some, in particular the α/β‐ and γ-type SASP, being the most abundant spore proteins (Setlow, 2007; Zhu and Stülke, 2018). These two types of SASP are degraded rapidly when spores germinate to provide amino acids for new protein synthesis. Notably if α/β-type SASP is not degraded, this causes the death of the outgrowing spore, as these proteins saturate spore DNA and if not degraded after germination, this will likely block transcription of much of the genome (Hayes and Setlow, 2001). Thus, it seems extremely unlikely that there will be any need for synthesis of proteins from these genes’ abundant mRNAs soon after spore germination is triggered. Indeed, recent work has shown that almost all abundant mRNAs are rapidly degraded following initiation of spore germination (Swarge et al., 2018). However, it is certainly possible that one or more spore mRNAs, in particular one of the most abundant ones, could direct synthesis of a protein or proteins that are essential for the development of the germinated spore. Indeed, there was one report that the malic enzyme encoded by *malS* was synthesized very early in *B. subtilis* spore germination, and even before the phase bright dormant spore turned phase dark due to excretion of CaDPA and cortex hydrolysis (Sinai et al., 2015). Evidence to support protein synthesis prior to the phase transition was principally in the form of an unexpected increase in fluorescence associated with a MalS-GFP fusion protein, coupled with Western blot data showing an apparent increase in abundance of the protein during this period. The same authors suggested that the concentration of malate in dormant spores was relatively high, in contrast to earlier studies and a more recent one (Setlow et al., 1977; Scott and Ellar, 1978; Korza et al., 2017), providing a readily available substrate with which to generate ATP for the germinating spore. A more recent study, employing analogous strains and a range of sporulation conditions, reported a similar increase in fluorescence associated with MalS-GFP during the early stages of germination (Swarge et al., 2020b). However, in contrast to results of Sinai et al. (2015), Western blot data in the Swarge et al. report indicated that MalS-GFP abundance remained stationary throughout germination. Indeed, mass spectrometry data, using a more sensitive approach than that employed by Sinai et al. (2015), convincingly demonstrates that synthesis of MalS does not begin until ~80 min after the completion of germination. The same authors suggest that the observed increase in MalS-GFP fluorescence reflects a change in the environment of the germinating spore core – most likely partial ingress of water from the environment promoting enhanced fluorescence from existing MalS-GFP – and is not associated with *de novo* protein synthesis. The observation that *malS* mRNA sits outside the top 1,000 most abundant transcripts detected within spores, at levels indicative of being present in only a small sub-set of the population and with a copy number averaging much less than 1/spore (Nagler et al., 2016; Korza et al., 2019), further supports the conclusions reported by Swarge et al. (2020b). Indeed, very recently published work conducted by the same group, this time combining an integrated transcriptomic and multi-faceted proteomic approach, permitting a hitherto unsurpassed level of resolution to these analyses in spores, robustly supports the idea that both transcription and protein synthesis start after the completion of germination (Swarge et al., 2020a).

There is also the question of when ATP becomes available in the developing spore, and as noted in section The Early Years, this appears to begin at most very late in spore germination, and perhaps not until this process is complete and spore core water content rises to the 80% of wet wt in growing cells. While all this evidence by no means conclusively proves that spore mRNAs are not saved to direct synthesis of some proteins when spores return to life in germination, these data certainly raise significant concerns about whether this could be the case.

It was the large dynamic range of the RNA-Seq data that led to a clearer picture of what spore mRNAs might do. Work done many years ago had indicated that as in most organisms, in growing cells of *B. subtilis* and a very closely related species, approximately 3% of their RNA is mRNA (Midgley, 1969; Brown and Coleman, 1975), and notably the uncharacterized RNA in *B. subtilis* spores termed “mRNA-like” was also ~3% of total RNA (Jeng and Doi, 1974). This number plus the RPKM values from RNA-Seq analysis of spore mRNAs in rRNA depleted spore RNA samples then allowed assignment of relative levels of all spore mRNAs. The other crucial number was the exact amount of RNA present in a single *B. subtilis* spore. This number, as well as the amount of spore DNA, was determined ~50 years ago in ^32^P-labeled *B. subtilis* and *B. megaterium* spores (Nelson and Kornberg, 1970a). Notably, although not known at that time, the value of *B. subtilis* spore DNA bp/spore determined in this report was almost identical to the length of the sequenced *B. subtilis* chromosome. In contrast, *B. megaterium* spores had slightly more than twice the DNA/spore than *B. subtilis* spores, as is now known to be consistent with *B. megaterium* making digenomic spores (Hauser and Karamata, 1992) and with a slightly larger chromosome than *B. subtilis*. Most importantly, the average values for RNA nt in *B. subtilis* spores, coupled with the assumption that 3% of spore RNA was mRNA as it is in growing cells, indicated that mRNAs in *B. subtilis* spores contributed an average of ~10^6^ nt/spore. Combining this latter number with the relative levels of spore mRNAs determined from RNA-Seq, and these mRNAs’ lengths adjusted for extra nt at the 5' and 3' ends, allowed calculation of the relative levels of these mRNAs needed to give the 10^6^ nt in *B. subtilis* spores (Korza et al., 2019). This analysis indicated that in *B. subtilis* there were only ~50 mRNAs present at ≥1 molecule/spore in populations, with many 100s present in <10% of the population. Notably, almost all of the abundant spore mRNAs are transcribed by RNA polymerase with σ^G^ or σ^F^ and encode proteins in spores. This suggests these mRNAs were those that were being made when the developing spore shut down, and they have been in suspended animation through dormancy.

While the above work certainly clarifies where the abundant and likely most significant spore mRNAs came from, they do not completely address the function of these mRNAs, as well as that of the many mRNAs present in only small fractions of the spore population. Might these be important in directing protein synthesis early in germination even if only for some spores? Ruling this out is most likely impossible. However, a simple experiment of leaving spores to sporulate for different times, and then analyzing their mRNA levels provided strong evidence that spore mRNAs, more specifically intact spore mRNAs, are not essential for at least *B. subtilis* spore germination and outgrowth (Camilleri et al., 2019). Thus RNA-Seq analysis on *B. subtilis* spores harvested after 2 days of sporulation gave approximately the same ~50 spore mRNAs at ≥1 molecule/spore, but spores allowed to sporulate on plates for 45–90 days at 37°C gave RNA from which levels of all spore mRNAs were decreased ≥95-fold, and rRNAs were significantly fragmented. Yet despite this loss of all intact spore mRNA and significant damage to rRNA, spore germination and return to growth were not appreciably affected. This result certainly rules out spore mRNAs as playing any role in directing protein synthesis when spores germinate, and suggests that these mRNAs main function is to serve as a reservoir of ribonucleotides for new RNA synthesis when spores germinate fully. As noted above, almost all the abundant spore mRNAs are rapidly degraded when spores germinate, but how this degradation takes place in the dormant spore is not clear, but it is not to ribonucleotides (Korza et al., 2016). Indeed, rRNA was also fragmented in spores after 45–90 days of sporulation, and at specific regions and into rather large fragments. Almost all mRNA from these aged spores is thus also almost certainly fragmented into pieces too small to be detected by RNA-Seq, but this will require further work to determine the fragment sizes of the degraded spore mRNAs. It is also possible that some small mRNA fragments play a regulatory role in gene expression, although there is currently no evidence relevant to this suggestion.

Probably the biggest question not answered to date is how so much mRNA from genes expressed in the mother cell shows up in dormant spores. Certainly, contamination in dormant spores’ outer layers has been thoroughly ruled out (Korza et al., 2019). However, there is known to be a feeding tube between the mother cell and forespore through which small molecules can pass to “feed” the developing spore (Figure 2). Perhaps fragments of mother cell mRNA can also pass into the developing spore as was previously suggested (Segev et al., 2012). Indeed, analysis of the coverage of RNA-Seq data for high and low abundance mRNAs in *B. subtilis* spores indicates that while coverage of reads of high abundance mRNAs is reasonably uniform, coverage of reads for low abundance mRNAs is somewhat less uniform, consistent with at least some of these mRNAs being in fragments. Indeed, direct evidence for the fragmented nature of at least some spore mRNAs was obtained recently by examining spore mRNAs for an NAD residue incorporated at mRNAs 5'-termini (Craft et al., 2020).

## Issues for the Future

Given all the material above, the most likely assumptions now about spore mRNA seem to be as follows: (1) this mRNA is not important for directing synthesis of proteins needed for spore germination or outgrowth and (2) the role of this mRNA is to serve as a ribonucleotide reserve for new RNA synthesis when spores resume macromolecular synthesis. The available data on spore ATP levels also make it extremely unlikely that there can be significant levels of new RNA synthesis in dormant spore populations. However, given the well-known heterogeneity in spore populations (Setlow et al., 2012, 2017), it is certainly possible that a small percentage of dormant spores could be active in RNA synthesis from time to time, and disproving such possible heterogeneity in dormant spore populations will be extremely difficult. Perhaps going forward it may become possible to conduct transcriptomic analyses on individual dormant spores. Alternatively, small fractions of spores within dormant populations have been observed to initiate germination in a seemingly stochastic manner that reflects phenotypic rather than genetic diversity (Paidhungat and Setlow, 2000; Sturm and Dworkin, 2015). It is plausible then that these “spontaneously” germinating spores account for detectable mRNA synthesis in otherwise dormant spore populations, particularly if sensitive assays are employed.

Another topic touched upon in this review is whether RNA and/or protein synthesis are needed for spore germination. Most, but not all evidence supports an answer of no to this question, with the lack of the inhibition of spore germination by inhibitors of RNA or protein synthesis seen by most workers being potentially definitive results. However, it is well-known that the dormant spore inner membrane (IM), at least of *B. subtilis*, is impermeable to charged compounds, and exhibits only very slow permeability to neutral lipophilic compounds and even water (Gerhardt and Black, 1961; Knudsen et al., 2016). Thus, it seems most likely that charged and hydrophilic inhibitors of macromolecular synthesis would not get into the spore core until after germination is complete, including the hydrolysis of the spore peptidoglycan cortex, when IM lipid mobility and permeability return to that of growing cells. Indeed, there is no direct evidence for uptake of inhibitors of RNA or protein synthesis into an intact dormant spore, including when various treatments have been used in hopes of increasing spore permeability (Tanimoto et al., 1996; Sinai et al., 2015). Thus, to make this inhibitor experiment definitive, new ways must be developed to ensure that inhibitors indeed are able to freely enter the spore core. The recent demonstration of the high apparent IM permeability of decoated, CaDPA-less spores which retain characteristics of dormant spores (Mokashi et al., 2020) suggests that these spores could be useful for this type of experiment.

The second major evidence against any essential involvement of macromolecular synthesis in spore germination is that ATP levels are almost non-existent in dormant spores, and appear to increase only after germination is complete (Setlow and Kornberg, 1970a; Scott and Ellar, 1978). While this result is potentially definitive in ruling out macromolecular synthesis’ involvement in spore germination, this experiment can be confounded to some extent by at least three factors – (i) the heterogeneity in germination rates between individual spores in populations (Setlow et al., 2017), so that curves for both germination and ATP accumulation are broadened significantly, (ii) completion of germination after CaDPA release requires hydrolysis of the spore cortex peptidoglycan, which leads to an increase in core water content to 80% of wet wt allowing full protein mobility in spores and thus metabolic activity; however, the latter increase in core water is not rapid and takes 10–15 min in *B. subtilis* (Camilleri et al., 2019), and perhaps there could be metabolic activity when core water content reaches 60% of wet wt, and (iii) analyses of the accumulation of ATP by germinating spores has always been measured by extraction of small molecules and subsequent analyses and it would be ideal to measure molecules such as ATP, NADH, etc., in individual germinating spores, as this would give much higher resolution for analyses of when ATP and other high energy compounds were generated in spore germination. Indeed, certainly our understanding of spore germination has been significantly increased by examining the germination of multiple individual spores (Setlow et al., 2017). It certainly seems possible that use of new technology to examine individual germinating spore properties could give further insight into events early in germination and with much better time resolution than we currently have.

Finally, one of the most puzzling findings to come from detailed analysis of mRNAs in dormant spores is that a number of these mRNAs are thought to be expressed only in the mother cell compartment of this sporulating cell under the control of the mother cell compartment-specific σ^E^ or σ^K^ factors for RNA polymerase. It is certainly possible that there is some small amount of either or both of these σ-factors in forespores, although expression of such mother cell proteins has not been observed, and indeed would seem to serve no purpose, especially for mRNAs that encode spore coat or exosporium proteins. Readthrough transcription from an upstream gene under control of a forespore promoter may also be a contributing factor here although it seems unlikely that such a mechanism would be relied upon to confer molecules with functional significance. Another possibility suggested ~8 years ago (Segev et al., 2012) is that mother cell mRNAs might migrate into the developing spore *via* the “feeding tube” through which the mother cell nurtures the forespore (Camp and Losick, 2008; Meisner et al., 2008; Camp and Losick, 2009; Crawshaw et al., 2014; Zeytuni and Strynadka, 2019). However, if these mRNAs were intact, this would again seem to raise the concern of expressing mother cell protein in the spore core. An obvious alternative is that the mother cell mRNAs are only fragments, which might even facilitate their passage through the feeding tube. Indeed, many spore mRNAs appear to be significantly fragmented as determined by both analysis of the coverage of reads along a gene (Camilleri et al., 2019; Korza et al., 2019), as well as isolation of 5'-modified regions of a few *B. subtilis* spore mRNAs and demonstration that 3'-regions of these mRNAs were not isolated with 5' ends (Craft et al., 2020). This analysis, as well as the mechanism of mRNA (as well as rRNA) hydrolysis in spores and which does not give rise to mononucleotides (Korza et al., 2016) are also subjects for further work, and will almost certainly give new and interesting information on what is going on inside developing spores, and even dormant spores themselves.

## Author Contributions

PS and GC contributed equally to the writing and preparation of this article. Both the authors contributed to the article and approved the submitted version.

### Conflict of Interest

The authors declare that the research was conducted in the absence of any commercial or financial relationships that could be construed as a potential conflict of interest.
